# Reducing unmet need for contraceptive services among youth in Homabay and Narok counties, Kenya: the role of community health volunteers – a qualitative study

**DOI:** 10.1186/s12913-021-06363-x

**Published:** 2021-05-01

**Authors:** Hermen Ormel, George Oele, Maryse Kok, Happiness Oruko, Beatrice Oluoch, Eefje Smet, Dorcus Indalo

**Affiliations:** 1 KIT Royal Tropical Institute, P.O. Box 95001, 1090 HA Amsterdam, The Netherlands; 2 Amref Health Africa, P.O. Box 30125, 00100 , Wilson Airport, Nairobi, Kenya; 3 Amref Flying Doctors Netherlands, Schuttersveld 9, 2316 XG Leiden, The Netherlands

**Keywords:** Community health volunteers, Community health workers, Contraceptive services, Family planning, Youth, Kenya

## Abstract

**Background:**

Access to contraceptive services is a cornerstone of human well-being. While Community Health Volunteers (CHVs) promote family planning in Kenya, the unmet need for contraceptives among youth remains high. CHVs seem to pay little specific attention to the contraceptive needs of the youth.

**Methods:**

We conducted a qualitative study exploring the role of CHVs in increasing access and uptake of contraceptive services among youth aged 18–24 years in Narok and Homabay Counties, Kenya. We undertook 37 interviews and 15 focus group discussions involving CHVs, youth, community members, community leaders, youth leaders and health programme managers. Data were recorded, transcribed, translated, coded and thematically analysed, according to a framework that included community, CHV and health system-related factors.

**Results:**

CHVs often operated in traditional contexts that challenge contraceptive use among unmarried female and male youth and young married couples. Yet many CHVs seemed to have overcome this potential ‘barrier’ as well as reigning misconceptions about contraceptives. While private and facility-based public contraceptive services were somehow available, CHVs were the preferred service provider for many youth due to ease of access and saving time and transport costs. This was influenced by varied perceptions among youth of CHVs’ knowledge, skills and attitudes regarding contraceptives and provider-client interaction, and specifically their commitment to maintain confidentiality.

**Conclusions:**

CHVs have the potential to increase access to contraceptives for young people, reducing unmet need for contraceptives. Their knowledge, skills and attitudes need strengthening through training and supervision, while incentives to motivate them and broadening the range of contraceptives they are allowed to offer should be considered.

**Supplementary Information:**

The online version contains supplementary material available at 10.1186/s12913-021-06363-x.

## Background

Improving access to preferred contraceptive methods is key to the well-being of women and men and benefits community health [1]. Yet there is a large unmet need for modern contraceptives in sub-Saharan Africa. About a quarter of couples who would like to space births do not use contraception. Providing timely contraception information and services to individuals and couples at health facilities or in the communities can reduce unmet need [2, 3].

Young people need special attention in terms of contraceptive services to promote sexual and reproductive health and avoid negative consequences of unprotected sex, such as unintended pregnancy, HIV and other sexually transmitted infections (STIs) [3].

In Kenya, 40% of married adolescent girls (15–19 years) use contraception; for sexually active unmarried girls of the same age group this is 50%. Percentages are higher for the same groups aged 20–24 years (54 and 70%, respectively). Preferred methods are injectables, implants and male condoms, which are mostly obtained from public services. Almost one in seven (14%) of girls aged 15–19 years have begun childbearing and about half (47%) of these births are unintended. Unmet need stands at 23 and 19% for the 15–19 and 20–24 age groups, respectively, as compared to 18% for all currently married women age 15–49 [4]. The relatively higher unmet need can be explained in part by challenges young Kenyans face in accessing contraceptive services, due to, inter alia, cultural notions that reserve such access to married couples with children [5], see contraceptive use a women’s responsibility [6] and emphasise the decision-making role of male partners [7]; problems of distance and (in) direct costs; and health service providers’ unwelcoming attitudes [8].

The Government of Kenya has reaffirmed its commitment to cater to the needs of adolescents and young people and to improve ‘existing service provision channels to provide accurate information and services on a wide range of contraceptive methods to capture diverse needs of adolescents’ [9].

Globally, community health workers (CHWs) are defined as health workers ‘carrying out functions related to health care delivery at household level; trained in some way in the context of the intervention, and having no formal professional or paraprofessional certificate or degree in tertiary education’ [10]. Many have come to see CHWs as ‘an integral component of the health workforce needed to achieve public health goals in low- and middle-income countries’ (LMICs) [11].

In Kenya, the Community Health Strategy (CHS) was launched in 2006 and later updated as the Strategy for Community Health 2014–2019 [12]. Under the CHS, government employed facility-based community health extension workers (CHEWs) to supervise a group of community health volunteers (CHVs) in every community health unit, each covering about 5000 people with an average of 1000 households. CHVs carry out promotive, preventive and some curative tasks in disease prevention and control, family health and hygiene and environmental sanitation [13, 14].

The 2012 WHO task-shifting guidelines in the field of maternal and newborn health suggest that certain interventions can be performed by CHWs, including contraceptive service delivery [15]. Evidence shows that, globally, CHW programmes can increase awareness and uptake of contraception, particularly where unmet need is high, access is low and geographic or social barriers to the use of services exist. CHWs are particularly important to reduce inequities in access to services, by bringing information, services and supplies to women and men in the communities where they live and work [16].

In Kenya, CHV tasks include family planning and adolescent reproductive health [13]. It includes promotion, discussion of all family planning methods and provision of basic counselling; addressing misinformation; and provision of selected contraceptive methods (mostly condoms) at community level [17].

Two pilot studies in Meru and Narok Counties concluded that CHV provision of injectables in Kenya was safe, acceptable, and feasible [18]. Some CHVs now provide injectables to clients who otherwise might not access facility-based services.

More attention is needed for awareness of and counselling about contraceptives among young people in Kenya. It is valid to explore the potential role of CHVs in bringing about improvements in youth access to contraceptives. Only a few studies have been conducted on the latter, with little documentation of how CHVs engage with youth. This study therefore aimed to explore the factors that influence the role of CHVs in access and uptake of contraceptive services by youth in Kenya.

## Methods

We undertook an exploratory qualitative study in Narok and Homabay counties, Kenya, to gain in-depth understanding of CHVs’ perspectives on providing services to youth and youth preferences for certain types of service providers.

Homabay and Narok counties represent diverse socioeconomic contexts while both exhibit high rates of teenage pregnancy. Both counties have implemented CHV programmes on family planning promotion while Narok joined the pilot that allowed CHVs to provide injectables. Within each county, two sub-counties and, within these, two community health units, were purposefully sampled, using urban/rural context and main ethnic population as criteria.

Semi-structured interviews (SSIs) and focus group discussions (FGDs) were conducted with four types of participants purposively sampled: young people 18–24 years, CHVs with minimum 6 months work experience, community members 35–49 years and key informants (community leaders, youth leaders, and Government and non-governmental organization (NGO) programme staff). Participants were selected based on sex, age, marital status and, where possible, ever having used contraceptives (young people) and work experience (CHVs). Young people were identified with the help of youth leaders, CHVs through CHEWs and community members through chiefs and programme administrators at the county and community levels.

We used SSIs to allow participants to be interviewed face-to-face in suitable venues like community (Chief, NGO-) offices and the County Health Management Team offices, exploring and probing sometimes sensitive topics while avoiding peer pressure that could result from group discussions. No others than those interviewed and the interviewers were present. Meanwhile, insight into group and institutional norms, behavior and use of health services was achieved by interactive, face-to-face FGDs, conducted in health facilities [19, 20].

Several topic guides for SSIs and for FGDs, tailored to the participant groups, were developed in English and translated to Swahili, specifically for this study (Supplementary file). Topic guides with youth addressed perceptions and perspectives on contraceptive needs and services. With CHVs, perceptions on youth behavior and needs and their own role as contraceptive service providers were discussed. The topic guides were tested in another sub-county than the study areas, after which they were slightly adjusted.

Data collection took place in April 2019 by an experienced and trained research team. Interviews and FGDs were done in English or Swahili, according to the preference of participants. Interviews and FGDs were conducted as presented in Table 1. Very few of the participants who were approached refused to collaborate; no repeat interviews were carried out.

**Table 1:** Overview of research methods and study participants

Method	Study population	County and number of participants, by sex	
		Narok County	Homabay County
*Semi-structured interviews (N = 37)*	Youth (18–24 years)	3 male, 2 female	4 male, 4 female
	CHVs	2 male, 1 female	2 male, 2 female
	Key informants type 1: community leaders	2 male	2 male
	Key informants type 2: youth leaders of selected youth groups	1 male, 1 female	1 male, 3 female
	Key informants type 3: managers (government, NGO)	2 male, 2 female	1 male, 2 female
*Focus group discussions*	Youth (18–24 years)	2 male groups, total 14 participants 1 female group, 8 participants	2 male groups, total 21 participants 2 female groups, total 22 participants
	CHVs	2 mixed groups, total 16 participants	2 mixed groups, total 23 participants
	Community members (35–49 years)	1 male group, 8 participants 1 female group, 9 participants	1 male group, 8 participants 1 female group, 12 participants

*CHV* community health volunteers, *NGO* non-governmental organisation

A field protocol was used to ensure adequate data collection. This involved, inter alia, delineating role division and internal communication, guidance on selection of study sites and study participants and a checklist on materials and equipment. Research assistants and supervisors held daily debriefings to discuss field notes and key findings. SSIs took up to 1 h to complete, FGDs up to 1.5 h; data collectors were selected and trained as regards their ability to translate questions into local dialect when necessary. Responses were digitally audio-recorded and transcribed in English. Researchers continued to recruit participants until the point of data saturation was reached and no additional, relevant knowledge emerged. Thirty-seven SSIs and 15 FGDs were conducted. Study participant key characteristics are summarized in Table 2.

**Table 2:** Amref study - Participant characteristics

		SSIs (***N*** = 20)								FGDs (11 FGDs, ***N*** = 104)							
		Youth				CHVs				Youth				CHVs			
		Homabay		Narok		Homabay		Narok		Homabay		Narok		Homabay		Narok	
		***N*** = 8		***N*** = 5		***N*** = 4		***N*** = 3		4 FGDs, ***N*** = 43		3 FGDs, ***N*** = 22		2 FGDs, ***N*** = 23		2 FGDs, ***N*** = 16	
**Participant characteristics**			Total		Total		Total		Total		Total		Total		Total		Total
Sex	*Female*		4	*2*		*3*		*1*		*22*		*8*		*18*		*9*	
	*Male*		4	*3*		*1*		*2*		*21*		*14*		*5*		*7*	
Age (years)	*Average*		23		22		49		42		21		23		38		38
Civil status	*Not married/in union*	*1*		*4*		*2*		*1*		*12*		*6*				*1*	
	*Married/in union*	*7*		*1*		*2*		*2*		*31*		*16*		*23*		*15*	
Education	*Primary incomplete*	*1*										*6*					
	*Primary*	*1*						*1*			11	*3*		*11*		*11*	
	*Secondary*	*5*		*4*		*3*					26	*6*		*12*		*5*	
	*Tertiary*	*1*		*1*		*1*		*2*			6	*7*					
Ethnicity	*Kalenjin*			*4*								*8*				*8*	
	*Kikuyu*					*1*						*1*					
	*Kipsigis*							*1*				*6*					
	*Kuria*											*1*					
	*Luo*		8			*3*				*43*		*1*		*23*		*1*	
	*Maasai*			*1*				*1*				*5*				*7*	
	*Teso*							*1*									
Religion	*Christian*		8		5	*4*				*43*		*21*		*23*		*16*	
	*Missing value*											*1*					
No. of children	*Average number*						5.6		4.3		1		1.5		4		5
	*No*	*2*		*4*													
	*Yes*	*6*		*1*													
Years of service as youth leader	Average		2		1.25												
	Range		1-4		0.1-2												
Years of service as CHV	Average						9		11.3								
	Range						2-15		8-16								

All transcripts were entered into an electronic qualitative data management and analysis software (Nvivo 11). A deductive approach, using pre-existing themes based on the study protocol and topic guides, was used to develop the coding framework. Emerging themes were added, inductively derived from the reading of an initial set of transcripts [21]. Transcripts were coded and data were further analyzed, ‘charted’ in themes and subthemes and summarized in narratives. While the coding was done by three experienced researchers, the full data analysis was conducted by the full team. Preliminary study findings were validated with health management teams in both counties in the first quarter of 2020. The outcomes were used to refine the study discussion and conclusions.

## Results

The findings of this study established that the role of CHVs in reducing unmet need for contraceptives among Kenyan youth was shaped by five key emerging themes: CHVs’ formal role regarding contraceptives, the influence of community norms and values, the role of CHVs as contraceptive providers for youth, CHV knowledge and skills and CHV motivation and incentives.

### CHV formal roles in providing contraceptive services

Our findings show that CHVs’ roles regarding contraceptive services entailed creating awareness, counselling, distribution of male condoms and referring to health facilities for any other contraceptive method. The exception were a limited number of CHVs in Narok, who have been trained as community-based distributors (CBDs) and who are providing injectables and hormonal pills, in addition to male condoms. All CHVs provide contraceptives to men and women of reproductive age alike to avoid unintended pregnancies and STIs.

In Homabay, CHVs, community members and key informants agreed that CHV contraceptive services were quite limited as regards provision of actual methods. One manager said:

“*They are not well motivated. Not all are trained on family planning services. And then, the commodities (...) they have not been given, so they are not well equipped.”* [Female manager, SSI, Homabay urban]

In Narok, after the introduction of the CHV-CBDs, congestion and workload at the health facilities had reduced as CBDs were now dispensing injectables and pills.

### CHVs vis-à-vis community norms and values

The majority of participants in both study areas agreed that culture and tradition often do not support the use of contraceptives. This view was especially strong in the Luo cultural context (Homabay), as illustrated by a young man:

“*I can say, the Luo culture doesn’t support family planning. A Luo believes that your manhood lies in getting many children of up to ten upwards. So when a person does family planning he cannot get the ten children, which is against the culture.”* [Single male youth, FGD/R10, Homabay rural]

Several participants across all study areas shared the view that women using contraceptives are seen as ‘promiscuous’. In Narok, on the other hand, a male CHV felt that there was nothing strange about the interaction between culture and family planning, as ‘in our culture we used to practice family planning, but without taking contraceptives’.

The majority of participants across all study sites agreed that (Christian and Muslim) ‘religion’ generally opposes contraceptive use by single youth and even by those who are married. Some however perceived that the role of religion is being overstated and ‘these days’ has less influence on decision-making on family planning.

“*The role of religion in my community, where I come from? They always advise people to use family planning, certain religions; but in another church people are told not to dare use family planning. It’s the leaders of the various denominations telling people what to do.”* [Married male youth, FGD/R4, Narok urban]

There was consensus among the majority of the youth that ‘culture’ and ‘tradition’ dictate that contraceptives are only for those who are married and already have children. The use of contraceptives by married couples who do not have children was frowned upon.

“*There is no woman, who is newly married, [who] is allowed to use family planning. She has to have a child in that homestead first. ( … ) That’s what [the family in-law] want.”* [Single female CHV, SSI, Homabay rural]

Many of the older participants in both counties did not agree with unmarried youth using contraceptives, considering this ‘immoral’. Yet others voiced different views, predominantly from Narok but also some from Homabay, identifying contraceptives as an important option for young people.

### CHVs as contraceptive providers for youth

In general, participants agreed that contraceptive services were equally offered to single youth as to the older, mostly married population. Some CHVs specifically expressed they were proud of their efforts to offer contraceptive services to youth. There were some suggestions that CHVs play a major role in the youth uptake of contraceptives, which is especially important given the ‘conservative’ family and cultural context.

“*The CHVs will really convince them. They know how to convince youths to go for it and they will go. Youths will go for family planning mostly because they [CHVs] have created the awareness … ”* [Married female youth leader, SSI, Homabay urban]

At the same time, some youth reported not being aware of CHVs offering contraceptive services. This can be explained since some CHVs reported they catered more to married than single youth. Some CHVs acknowledged they did not offer contraceptive services to single youth, given their own religious (mostly Christian) values. A female County manager flagged the need to address this:

“*The cultural beliefs and values … [CHVs] need to get from us. For example, as health care workers, we also have our values, but we need to tell them that ‘you leave your value aside then you handle the client the way the client is.’*” [Female manager, SSI, Homabay rural]

Many youth participants maintained that CHVs were their contraceptive providers of choice. CHVs were perceived as more easily accessible, saving time and transport costs, and taking more time to interact. This perspective was more pronounced among single youth yet also supported by several married youth.

### CHVs’ knowledge and skills

Preference for CHVs by youth often presupposed a perception of ‘similar quality’ of services as compared to those provided by facility-based health workers. Some youth indeed perceived this or simply compared CHVs’ knowledge to their own lack of knowledge:

*“[CHVs] know more about family planning and we just know a little about it. So they continue to open your mind on family planning with the information that they add on us, so my heart just loves them.”* [Married female youth, SSI, Homabay rural]

Few youth as well as other participants perceived that facility services were of better quality, as they questioned the knowledge and skills of CHVs. As one Narok single female youth leader put it, CHVs ‘don’t give information that is sufficient or reliable’.

CHVs themselves, across both counties, reported that training was either an enabler or disabler in the provision of contraceptive services. Those who did not have adequate training expressed the need for refresher training:

*“Personally I feel like I have not yet gotten proper training. And so a young person may ask me a technical question and I fail to know what to answer to it.”* [Married female CHV, FGD/R11, Homabay rural]

Some health workers, especially in Homabay where the CBD model has not been introduced, were not in favour of CHVs taking up new roles regarding contraceptive services (‘task shifting’). They felt that CHVs, even when trained, would not be ready to deal with contraceptive side effects, among others.

We found that confidentiality was very important, especially to single youth. Some youth found that CHVs could be trusted to keep confidentiality. Other youth doubted CHVs’ ability to maintain confidentiality and therefore were not confident to seek contraceptive services from CHVs.

“*Me going to a CHV I think sometimes that confidentiality may not be maintained. If I go for the contraceptive today and I go and share with a friend and the following day I go again, she might see me as someone who is highly promiscuous. So I would rather go to the chemist.”* [Married male youth, FGD/R8, Homabay urban]

CHVs themselves maintained that they keep whatever information youth share with them confidential.

### CHV motivation and incentives

A number of CHVs in both counties expressed an intrinsic motivation of love for voluntary work, ‘doing a service to God’ and desire to assist fellow community members. This is also how some community members saw their role:

“*CHVs are volunteers, and volunteers get nothing. They have only given themselves in to work. CHVs are the level one and they teach people from the heart for free.”* [Married female community member, FGD/R4, Narok rural]

CHVs also referred to extrinsic motivational factors, in terms of: the satisfaction of aiding the well-being of individuals and the community at large; the recognition and respect received; and developing new knowledge and skills. Some felt they were helping to address maternal mortality, reduce child malnutrition or teenage pregnancies, and that community members were able to save time and money because of their work.

*“When there is a meeting with the chief, you get the chance and courage to address the people. Also I get recognition from the community who refer to me as ‘doctor.’”* [Female CHV, FGD/R8, Narok rural]

“*This job has helped me a lot, I’ve been given more knowledge ( … ). It has made me somebody. ( … ) Whenever I see someone has followed my advice and succeeds, that’s what motivates me a lot.”* [Single female CHV, SSI, Homabay rural]

Intrinsic and certain extrinsic motivational factors constitute incentives that push CHVs to do the work they do. At the same time, nearly all CHVs and a number of other participants indicated that motivation is often negatively affected due to the absence of financial or other material incentives. Many CHVs indicated they felt they deserved some form of financial ‘incentive’ in return for the commitment, many hours of service and hard work. Some thereby also referred to the needs of their own family:

*“I would like the government to give us some motivation in order for the work to be done effectively. ( … ) The kind of motivation we need is money. Because with this work, I will end up having walked around till evening and have nothing to bring back to my children. So this has a challenge because it is fulltime volunteer work.”* [Married male CHV, FGD/R8, Narok urban]

A few CHVs and one County manager expressed that financial rewards had been promised since some time ago but never materialized, causing frustration. Apart from financial compensation, many mentioned the importance of varying types of material incentives, such as umbrellas, gumboots, flashlights and transport such as bicycles. These would strengthen CHV performance, including on contraceptive services.

Other CHVs, across both counties, felt demotivated due to the lack of a ‘career perspective’, like moving into a paid job.

*“But what I do not like, we are stuck at being volunteers with a lot of work. We see no future of becoming CHEWs. So there is no payment.”* [Married female CHV, FGD/R2, Home Bay rural]

## Discussion

This study intended to explore the factors influencing the role of CHVs in increasing access and uptake of contraceptive services among the youth aged 18–24 in Narok and Homabay Counties in Kenya.

The role of Kenyan CHVs in reducing unmet need for contraceptives among Kenyan youth was shaped by five key emerging themes, with following results. First, the formal role of CHVs regarding contraceptives is limited. Second, community norms and values can be challenging but do not stand in the way of an expanded role of CHVs to reduce unmet need. Third, CHVs are the preferred contraceptive providers of many youth. Fourth, CHVs knowledge and skills need attention, including issues around confidentiality. Fifth, CHVs lack financial or material incentives to better perform their role.

As for the first theme, participants had similar views on the essentially preventive and promotive nature of CHV roles. They agreed that the role of CHVs regarding contraceptive services was limited, as most lacked training, motivation and commodities. This was perceived differently in Narok, because a number of CHVs had been trained to provide a broader range of contraceptives [18]. In their systematic review of 56 studies on the effectiveness of CHWs’ roles as providers of contraceptive services in LMIC, Scott et al. concluded that CHWs were able to increase the uptake of modern contraceptives as well as improve levels of contraceptive-related knowledge and attitudes [22]; others reached a similar conclusion for Sub-Sahara Africa and beyond [15, 23].

Many Kenyan CHVs saw culture and tradition as mostly not supportive of contraceptive use, often linked to valuing children as a symbol of wealth; this was also observed in another Kenyan study [24]. Such beliefs were, in turn, intertwined with religious values, which according to most oppose contraceptive use among single youth [25], but also among married youth without children. Although most study participants agreed that CHVs in principle served unmarried youth at par with married couples, few CHVs refused to cater for unmarried youth out of personal religious principles, reflecting findings in Tanzania [25]. While this is somehow understandable, as CHVs are part of and originate from the communities they serve and as such share similar cultural views, it points to the need for improved recruitment processes and/or training on the separation of personal and professional values. The same applies to professional health workers [26]. However, CHW (training) programmes globally seldom pay systematic attention to reflection on norms and values, leading to CHW having challenges in dealing with culturally sensitive issues [27].

Most CHVs felt that ‘conservative’ cultural norms and values should not prevent someone from accessing and using contraceptives, in view of the important benefits. This can be seen as an example of how some CHVs not only contributed to managing the sometimes perceived conflict between ‘culture’ and ‘family planning’, but also to the empowerment of beneficiary communities. That said, CHVs in both counties were regarded as important facilitators of youth uptake of contraceptives in a ‘conservative’ social environment. One global systematic review also concluded that several community-based interventions combined, including home visits by CHWs, increased uptake of contraceptives among young married couples [20].

Many CHW programmes worldwide have some form of commitment to this concept [26]. One study found that, in order to enable CHWs to play their role as ‘agents of change’, CHWs’ own empowerment is a precondition. Yet this is faced with barriers related to feelings of non-appreciation by communities, health workers or both, and feelings of being undervalued and ‘not in control’ [27].

Youth in this study perceived CHVs as more easily accessible than other health workers, including taking more time to interact. Similar youth preferences were observed in Malawi, where an experimental study assessed the relationship between service features and 15–24-year-old youth contraceptive provider preferences. Findings globally were that community-based services were preferred over services that were facility-based. CHW services were assessed as an effective way to improve access to and uptake of contraceptives among rural youth [28, 29].

The potential for CHVs to better meet youth’s unmet need for contraceptives seemed undermined by perceptions that CHVs lack essential knowledge and skills. This was also observed in another Kenyan study, noting hesitation among women to use CHV services due to concerns about quality of services [30]; the need for ongoing training of CHWs is also documented more generally [11, 31].

Some interviewed CHVs confirmed the need for further training on contraceptive methods, side effects and counseling skills. A 2014 study in South Kivu province, DRC, on the interaction between CHWs and youth clients regarding sexual and reproductive health related services, showed that many CHWs reported low confidence in having sufficient knowledge to communicate with youth [32].

The issue of maintaining confidentiality was important for youth, especially for ‘sensitive’ topics like sexuality and contraceptives, which are associated with various taboos and stigma [28, 33]. While some study participants felt that CHVs could be trusted, others questioned this. International literature emphasizes the need for trusting relationships between CHW and their clients, as well as the community at large and enhanced CHWs’ interpersonal skills regarding confidentiality [34, 35]. Once confidentiality is established, the CHV would be the most preferred service provider for many young study participants [36].

Participants identified various intrinsic and extrinsic motivational factors for the work performed and many perceived that CHV motivation is often affected due to the lack of financial or material incentives. Our findings confirm what other studies found globally: in all kinds of contexts and despite their intrinsic motivation, voluntary CHWs often also expect some form of financial compensation; this is especially the case when workload expands [37, 38]. The World Health Organization recommends that CHWs should be remunerated in line with tasks, training and hours of service per week [35]. The current Kenyan policy recommends that CHVs should be paid USD20 per month [12]; but it does not indicate the source of funds and how it would be allocated. In the recent context of devolved governance, some county governments are in the process of legislating for CHVs to be recognized and payed a stipend under the County Act of Assembly.

Recent studies found that financial or material incentives do not necessarily always lead to improved motivation. Enabling factors should also be present, including resources to perform the work required (supplies, transport), supportive supervision and training and professional development opportunities [31, 35, 39–41]. To different degrees, each of these issues were flagged by our participants as needing attention to improve CHV motivation and performance.

This study has several strengths and limitations. Data analysis and discussion of findings was done involving a multi-disciplinary group of researchers. The qualitative approach served the purpose of creating better understanding of the what, why and how of community, youth and CHV perspectives. Participants were from four sub-counties across two counties, selected for certain differences but also similarities. This implies that results do not represent the diversity of all Kenyan counties. Selection bias is a risk inherent to the selected methods, only partially compensated for by our sampling strategy. Potential researcher bias was addressed by having teams of two doing data collection and having yet another researcher transcribing the recorded data. The sensitive nature of topics addressed implies a risk of bias towards social desirable answers, which we tried to deal with by training of data collectors and triangulating methods and participant types.

## Conclusions

CHVs constitute a major potential to increase access to contraceptives for youth, reducing unmet need. However, this study found that CHVs’ roles as community-based distributers of contraceptives are as yet limited, which calls on the Ministry of Health to enforce the 2017 task-shifting guidelines. Many young people prefer CHVs as contraceptive service providers above facility-based health workers in the public or private sector. One precondition is that a trusting and confidential relationship is established between CHVs and their young clients. For this reason, and for building essential knowledge and skills to provide higher-quality contraceptive services in ‘conservative’ social settings, the Ministry of Health and other stakeholders should provide CHVs with improved training. To further facilitate CHVs’ role in the provision of contraceptive services, national and local government bodies and relevant stakeholders should offer financial compensation and ensure other enablers such as transport and supportive supervision are instrumental. It is also urgent that CHVs, often seen as important ‘agents of change’, get assistance from other stakeholders, such as religious leaders, to address prevailing cultural and religious norms and create more understanding and support for contraceptive services at community level, including for youth.

## Supplementary Information

**Additional file 1.**

## Data Availability

The datasets generated and analysed during the current study are available from the country study authors on reasonable request; contact details are available through the corresponding author.

## Abbreviations

CBD: Community-based distributor
CHEW: Community health extension worker
CHV: Community health volunteer
CHW: Community health worker
FGD: Focus group discussion
LMIC: Low and middle-income country
NGO: Non-governmental organisation
SSI: Semi-structured interview
