# Supplementary material for: Reducing unmet need for contraceptive services among youth in Homabay and Narok counties, Kenya: the role of community health volunteers – a qualitative study
Source: BMC Health Serv Res. 2021 May 1;21:405. doi: 10.1186/s12913-021-06363-x (PMC8088547; doi:10.1186/s12913-021-06363-x)
Supplement: Supplementary file 1 — Additional file 1. [file 12913_2021_6363_MOESM1_ESM.docx]

**Supplementary file: Data collection instruments**

FGD-1 - Topic guide for youth (M/F, 18-24yrs, married or in union/not married or in union, associated with a youth group)

| CODE |  |
| --- | --- |
| Date |  |
| Moderator |  |
| Recorder |  |
| Transcriber |  |
| Venue/location (county, CU) |  |
| Start time |  |
| End time |  |
| Duration |  |
| General comments by the moderator and recorder | How was the process? Was it difficult/easy to manage? Why? Were participants (un) comfortable? Why? |

Assign each of the participants a number (1-xx) and after reading the consent form and getting their consent, fill in the following table by speaking to them one-to-one, before the start of the FGD:

| Partici-pant number | Age  -in whole years | Sex  -M  -F | Marital status –married/in union  -unmarried/ not in union | No. living children | Education  -none  -up to completed primary  -up to completed secondary  -higher educ | Ethnicity | Religion |
| --- | --- | --- | --- | --- | --- | --- | --- |
| 1 |  |  |  |  |  |  |  |
| 2 |  |  |  |  |  |  |  |
| 3 |  |  |  |  |  |  |  |

Start the recording and focus group discussion. Introduce the context and topic of research. Use the following questions (numbered but no need to necessarily follow in this order and probes (not always but if needed; bulleted):

Questions

1. **Family planning general**
2. When we talk about Family Planning, how do you understand it? What is Family Planning?
3. What can be reasons to use FP methods?
   - Probe for issues: STIs, HIV, pregnancy prevention (postponing, spacing, limiting)
4. What types of FP methods do you know/have you heard about?
5. Do youth [we mean 18-24 years] like you sometimes also use FP methods? Why?
   - Probe: Prevention of pregnancy, HIV, STIs?
6. What methods do youth prefer? Why?
7. Who decides on the use of a method?
   - Probe: the girl/woman, the boy/man, together?
   - Probe: Is anybody else involved in decision-making on the use of FP by young people, e.g. parents, family-in-law, others?
8. **Providers, locations and services**
9. Where and from whom do the youth normally get FP methods from?
   - If needed, probe for facility and person: local government clinic (nurse, other?), local pharmacy/drug seller, private clinic, other
10. Why do youth go to that specific facility/provider for these services?
    - Probe for reasons
11. When youth go for FP services, what type of service are they looking for?
    - Probe: general information on method(s), counselling on what is best for their situation, obtaining the method of their choice
12. Are FP services also brought closer to youth? If yes: by whom and how frequent?
    - If needed probe: are these services also available in the community, outside the clinic?
    - Note - if CHV is mentioned: consider continuing with those questions first before returning here
13. **Potential barriers**
14. Regarding available FP services: what are youth happy about regarding the performance of service providers, and what are they not happy about?
15. Do youth get the method of their choice every time they go for it? If not, do you know what are the reasons for unavailability of the method? Then what do youth do? How long does it last before the method they need becomes available again?
16. How far away is the location of the FP service from where youth reside? Is this convenient? How do youth go there? Do youth have to pay for transport?
17. What are the opening hours of the locations youth normally get the FP service from? Is this convenient?
18. Do youth have to pay for the methods when they get them? Is it easy to afford? Is the cost related to obtaining FP services/methods sometimes a problem?
19. **CHVs as service providers**
20. Do CHVs also offer FP services? Do you know youth who have used FP services from CHVs? Like information, counselling, obtaining FP methods?
21. If Yes: What do you think about the FP services by CHVs? Is it the same as for other providers, or not as good, or better? Why?
22. What is different?
    - Probe: e.g. choice of methods? The information provided by the person? Quality of service [and what does that mean]?
23. Are there advantages of CHVs providing FP services? Do you see any disadvantages?
24. Would you make use of FP services from a CHV? Why yes/no?
25. What can be done to improve FP services offered by CHVs?
    - Probe:
      - Did they receive proper training or need more?
      - Do they get enough support from health workers or need more/different support?
      - If CHVs don’t know or can’t help, do they refer to the nurse?
      - Are CHVs well-motivated for their job and for providing FP services?
26. **Norms and values**
27. Would you say that there are certain norms or cultural beliefs in your community about using FP methods?
28. What are some examples of such norms and values?
    - Probe:
      - What about youth who are not married or in union, are they supposed to use FP methods?
      - What about couples married or in union who don’t have children, are they supposed to use FP methods?
      - And couples with already a few children?
29. What do you yourself think of such norms and values? Do you sometimes feel different about this or do you mostly agree?
30. What role does religion play when it comes to FP services in your community?
    - Probe for what the religious leaders tell say about FP
31. In your community and culture, are there norms and customs that are likely to support or hinder use of FP services? Give examples?
32. In families or the community, are there people that help or hinder, when youth want to use FP methods?
    - Probe for examples; support or hindrance from parents, partners, community leaders, clinic staff, etc.
    - Probe: are friends likely to influence a youth’s decision to take up FP services? How, who, when?
33. Have you ever come across FP topics being discussed in the media? Are such discussions likely to influence youth decision-making to use FP services?
34. How do you think the community can be more involved in providing family planning services to the youth?

FGD-2: Topic guide for focus group discussion with community health volunteers (M/F, trained on FP/RMNCH)

| CODE |  |
| --- | --- |
| Date |  |
| Moderator |  |
| Recorder |  |
| Transcriber |  |
| Venue/location (county, CU) |  |
| Start time |  |
| End time |  |
| Duration |  |
| General comments by the moderator and recorder | How was the process? Was it difficult/easy to manage? Why? Were participants (un) comfortable? Why? |

Assign each of the participants a number (1-xx) and after reading the consent form and getting their consent, fill in the following table by speaking to them one-to-one, before the start of the FGD:

| Partici-pant number | Age  -in whole years | Sex  -M  -F | Marital status –married/in union  -unmarried/ not in union | No. living children | Education  -none  -up to completed primary  -up to completed secondary  -higher educ | Lives where  -county  -CU | Active as CHV since how many years  -in whole years |
| --- | --- | --- | --- | --- | --- | --- | --- |
| 1 |  |  |  |  |  |  |  |
| 2 |  |  |  |  |  |  |  |
| 3 |  |  |  |  |  |  |  |

Questions

**Role as CHV**

1. How were you recruited as community health volunteers? Who initiated contact; how did you come to learn about it?
2. Where were you recruited: public Barasa, health facility, by CHC
3. What were the selection criteria: education background, age, gender, religion, other

- Probe: training on FP (knowledge and skills)?
- Probe: capacity to engage with the community on FP and SRH issues?

1. How much time do you spend as CHV every day/week? What are your daily tasks?
2. What attracted you to this job?
3. What do you like and what don’t you like about the job?
   - Probe: support from community, support from the CHMT and facility health workers, the workload

**Youth and FP services in general**

1. Do youth [= 18-24 years] sometimes use FP methods? Why?
2. What type of methods do youth prefer and why?
3. Where and from whom do the youth normally get FP methods from?
   - If needed, probe for facility and person: local government clinic (nurse, other?), local pharmacy/drug seller, private clinic, other
4. Why do youth go to that specific facility/provider for these services?
   - Probe for reasons
5. When youth go for FP services, what type of service are they looking for?
   - Probe: general information on method(s), counselling on what is best for their situation, obtaining the method of their choice
6. What are the issues that make the youth consider going for the FP services or not?
   - Probe for:
     - Enabling environment that is or is not friendly to the interest of the youth
     - Youth risk of being stigmatized/isolated by peers
     - Societal/community norms and values (community see youth as not qualified to access community services)
     - Individual norms and beliefs (community traditional beliefs)

**CHV role in FP services in general and for youth**

1. What is your role as CHV in the provision of FP services in the community? What kind of FP services do you offer?
   - Probe for: general information on method(s), counselling on what is best for their situation, FP methods
2. To what groups do you offer these services?
   - First wait for answers, ask: ‘any other groups’?
   - Then if needed probe for: married couples? Single women/men? Youth? Other?
3. What type of FP methods do you offer to youth? Are these the methods that youth want?
4. What makes you as CHV more relevant to provide FP services to the youth, both male and female?
   - Wait for answers, ask ‘what else’
   - Then if needed, probe for other CHV characteristics: sex, age, marital status, education level, training receive don FP (knowledge and skills)
   - Probe for how CHV services are different/better than services in facilities elsewhere?
5. What do you consider as helpful or hindering factors for a CHV to provide FP services to youth?
   - Wait for spontaneous answers
   - Then probe for provider-side issues:
     - Range/types of FP methods offered in general
     - Any stock outs of preferred FP methods
     - Availability of educational materials for FP for reference
     - Distance, transport
     - Cost
     - Other?
   - And probe for client/community-side issues:
     - Support from community leaders
     - Support from parents
     - Interest from youth themselves
     - Other?
6. What issues affect your support to FP services for youth? What support do you require to deliver more effective FP services to the youth?
   - Wait for spontaneous answers, then if needed probe for:

Health service side

- Training on FP services
- Availability/access to the FP commodities
- Supervision from Health Care workers (CHEWs, nurses)
- Task clarity on provision of FP services by CHVs
- Mentorship and coaching
- Feedback on services provided by CHVs

Clients/community side

- Community support/norms
- Other?

**Norms and values**

1. Do you feel it is good to offer FP services to youth? Why yes/no?
2. Would you say that there are certain norms or cultural beliefs in your community about using FP methods? What are some examples of such norms and values?
   - Probe:
     - What about youth who are not married or in union, are they supposed to use FP methods?
     - What about couples married or in union who don’t have children, are they supposed to use FP methods?
     - And couples with already a few children?
3. What do you yourself think of such norms and values? Do you sometimes feel different about this or do you mostly agree?
4. In your community and culture, are there norms and customs that are likely to support or hinder use of FP services? Give examples?
5. And for youth: are there norms and customs that are likely to support or hinder use of FP services by youth?
6. Do you feel prepared and comfortable to engage with the community on FP and SRH issues

FGD-3 – Topic guide community members (M/F, 35-49yrs)

| CODE |  |
| --- | --- |
| Date |  |
| Moderator |  |
| Recorder |  |
| Transcriber |  |
| Venue/location (county, CU) |  |
| Start time |  |
| End time |  |
| Duration |  |
| General comments by the moderator and recorder | How was the process? Was it difficult/easy to manage? Why? Were participants (un) comfortable? Why? |

Assign each of the participants a number (1-xx and after reading the consent form and getting their consent, fill in the following table by speaking to them one-to-one, before the start of the FGD:

| Partici-pant number | Age  -in whole years | Sex  -M  -F | Marital status –married/in union  -unmarried/ not in union | No. living children | Education  -none  -up to completed primary  -up to completed secondary  -higher educ | Lives where  -county  -CU | Profession | Time lived in community  -years |
| --- | --- | --- | --- | --- | --- | --- | --- | --- |
| 1 |  |  |  |  |  |  |  |  |
| 2 |  |  |  |  |  |  |  |  |
| 3 |  |  |  |  |  |  |  |  |

Start the recording and focus group discussion. Introduce the context and topic of research. Use the following questions (numbered but no need to necessarily follow in this order and probes (not always but if needed; bulleted):

Questions

**A. Family planning general**

1. When we talk about Family Planning, how do you understand it? What is Family Planning?
2. What can be reasons for members of the community to use FP methods?
   - Probe for issues: STIs, HIV, pregnancy prevention (postponing, spacing, limiting)
3. What types of FP methods do you know/have you heard about?
4. Do youth [**we mean 18-24 years**] sometimes also use FP methods? Why?
   - Probe: Prevention of pregnancy, HIV, STIs?
5. What methods do youth prefer? Why?
6. Who decides on the use of a method?
   - Probe: the girl/woman, the boy/man, together?
   - Probe: is anybody else involved in decision-making on the use of FP by young people, e.g. parents, family-in-law, others?

**B. Norms and values**

1. Do you feel it is good to offer FP services to youth? Why yes/no?
2. Would you say that there are certain norms or cultural beliefs in your community about using FP methods?
3. What are some examples of such norms and values?
   - Probe:
     - What about youth who are not married or in union, are they supposed to use FP methods?
     - What about couples married or in union who don’t have children, are they supposed to use FP methods?
     - And couples with already a few children?
4. What do you think of such norms and values? Do you agree or sometimes feel different about this?
5. What role does religion play when it comes to FP services in your community?
   - Probe for what the religious leaders tell say about FP
6. In your community and culture, are there norms and customs that are likely to support or hinder use of FP services? Give examples?
7. And for youth: are there norms and customs that are likely to support or hinder use of FP services by youth?
8. In families or the community, are there people that help or hinder, when youth want to use FP methods?
   - Probe for examples; support or hindrance from parents, partners, community leaders, clinic staff, etc.
   - Probe: are friends likely to influence a youth’s decision to take up FP services? How, who, when?
9. Do you think the community should be more involved in making it possible for family planning services to be provided to the youth?

**C. Providers, locations and services**

1. Where and from whom do the youth normally get FP methods from?
   - If needed, probe for facility and person: local government clinic (nurse, other?), local pharmacy/drug seller, private clinic, other
2. Why do youth go to that specific facility/provider for these services?
   - Probe for reasons
3. When youth go for FP services, what type of service are they looking for?
   - Probe: general information on method(s), counselling on what is best for their situation, obtaining the method of their choice
4. What are the issues that make the youth consider going for the FP services or not?
   - Probe for:
     - Enabling environment that is either or not friendly to the interest of the youth
     - Youth risk of being stigmatized/ isolated by peers
     - Societal/community norms and values (community see youth as not qualified to access community services)
     - Individual norms and beliefs (community traditional beliefs)
5. Are FP services also brought closer to youth? If yes: by whom and how frequent?
   - If needed probe: are these services also available in the community, outside the clinic?
   - Note - if CHV is mentioned: consider continuing with those questions first before returning here

**D. Potential barriers**

1. Regarding available FP services: what are youth happy about regarding the performance of service providers, and what are they not happy about?
2. Do youth get the method of their choice every time they go for it? If not, do you know what are the reasons for unavailability of the method? Then what do youth do? How long does it last before the method they need becomes available again?
3. How far away is the location of the FP service from where youth reside? Is this convenient? How do youth go there? Do youth have to pay for transport?
4. What are the opening hours of the locations youth normally get the FP service from? Is this convenient?
5. Do youth have to pay for the methods when they get them? Is it easy to afford? Is the cost related to obtaining FP services/methods sometimes a problem?

**E. CHVs as service providers**

1. Do you have community health volunteers (CHVs) in your community?
2. What is their role, what services do they offer?
3. How important are their services, how would you rate their services?
4. Do CHVs also offer FP services? Do community members use CHVs for FP services? Like information, counselling, obtaining FP methods?
5. Do youth also use FP services from CHVs? If not, why?
6. If Yes: What do you think about the FP services by CHVs? Is it the same as for other providers, or not as good, or better? Why? What is different?
   - Probe: e.g. choice of methods? The information provided by the person? Quality of service [and what does that mean]?
   - Probe to know whether they think the youth prefer being served by the males of female
7. Are there advantages of CHVs providing FP services? Do you see any disadvantages?
8. And for youth: are CHVs the right people to provide FP services to youth?
9. If you needed FP services, would you go to a CHV? Why yes/no?
10. What can be done to improve FP services offered by CHVs?
    - Probe:
      - Did they receive proper training or need more?
      - Do they get enough support from health workers or need more/different support?
      - If CHVs don’t know or can’t help, do they refer to the nurse?
      - Are CHVs well-motivated for their job and for providing FP services?
      - Other?

SSI-1: Topic guide for interviews with youth (M/F, 18-24yrs, married or in union/not married or in union, FP users (current or past)/non-users, (peri)urban/rural)

| CODE |  |
| --- | --- |
| Date |  |
| Moderator |  |
| Recorder |  |
| Transcriber |  |
| Venue/location (county, CU) |  |
| Start time |  |
| End time |  |
| Duration |  |
| General comments by the moderator and recorder | How was the process? Was it difficult/easy to manage? Why? Were participants (un) comfortable? Why? |

After reading the consent form and getting their consent, fill in the following table:

| Job title |  |
| --- | --- |
| Years of service |  |
| Age – in whole years |  |
| Sex -M/F |  |
| Marital status  –married/in union  -unmarried/ not in union |  |
| No. living children |  |
| Education  -none  -up to completed primary  -up to completed secondary  -higher educ |  |
| Ethnicity |  |
| Religion |  |

Only fill in after the end of the interview, when the related questions have been asked:

| FP use:  -user (present or past)  -non-user (never) |  |
| --- | --- |

**Perceptions and preferences of youth on access to FP services from CHVs and other providers**

Questions

1. What do you understand by the term Family Planning service?
2. What can be reasons for people to use FP methods?

- Probe for benefits, risk: STIs, HIV, pregnancy prevention (postponing, spacing, limiting)

1. What types of FP methods do you know/have you heard about?
2. Are you currently using or have you ever used any family planning service before?

**🡺IF YES [‘IF NO’ see further below]**

**A. Family planning in general**

1. What is the reason for you to use FP?
2. What FP method(s) do you prefer to use? Why do you prefer this particular method?
   - - Probe for benefits, risk: STIs, HIV, pregnancy prevention/spacing/limiting)
3. Who decides on the use of a method?
   - Probe: you, your partner, together?
4. Is anybody else involved in the decision on the use of FP?
   - Probe for role: father, mother, father/mother-in-law, others?

**B. Providers, locations and services**

1. Whom and where do you normally get these FP services from?
   - Why do you go there/to that person?
2. Is this fine for you or ewould you actually prefer to get these FP services from someone else or somewhere else?
   - If yes, from whom and where?
     - If needed, probe for facility and person: local government clinic (nurse, other?), local pharmacy/drug seller, private clinic
   - Why do you prefer this place/person to get these methods?
   - Note: if they indicate to prefer CHV, then consider jumping to the CHV section
3. When you go, what service are you looking for
   - Probe: general information on method(s), counselling on what is best for my/our situation, obtaining the method of my choice
4. Are these services also brought closer to you? If yes: by whom and how frequent?
   - If needed probe: are these services also available in the community, outside the clinic?
   - Note - if CHV is mentioned: consider continuing with those questions first before returning here

**C. Potential barriers**

1. Regarding available FP services: what are you happy about regarding the performance of service providers, and what are you not happy about?
2. Do you get the method of their choice every time you go for it? If not, do you know what are the reasons for unavailability of the method? Then what do you do? How long does it last before the method you need becomes available again?
3. How far away is the location of the FP service from where you reside? Is this convenient? How do you go there? Do you have to pay for transport?
4. What are the opening hours of the locations you normally get the FP service from? Is this convenient?
5. Do you have to pay for the methods when you get them? Is it easy to afford? Is the cost related to obtaining FP services/methods sometimes a problem?

**D. CHVs as service providers**

1. Do CHVs also offer FP services? Have you used FP services from CHVs? Like information, counselling, obtaining FP methods?
2. If Yes: What do you think about the FP services by CHVs? Is it the same as for other providers, or not as good, or better? Why?
3. What is different?
   - Probe: e.g. choice of methods? The information provided by the person? Quality of service [and what does that mean]?
4. Are there advantages of CHVs providing FP services? Do you see any disadvantages?
5. Will you continue to make use of FP services from a CHV? Why yes/no?
6. What can be done to improve FP services offered by CHVs?
   - Probe:
     - Did they receive proper training or need more?
     - Do they get enough support from health workers or need more/different support?
     - If CHVs don’t know or can’t help, do they refer to the nurse?
     - Are CHVs well-motivated for their job and for providing FP services?

----------

🡺IF NO

1. Why not?
2. Can you imagine that at some point in your life you will use an FP method?
   - If no – why not?
   - If yes:
     - what method might then have your preference? Why?
     - Would you know where to go to obtain information and the method? [probe all providers/locations]
       - If they don’t mention CHV:
         - would you also consider going to a CHV? Why yes/no?
         - CHVs can also provide FP services. Are there advantages of CHVs providing FP services? Do you see any disadvantages?
       - If they mention CHV:
         - What do you think about the FP services by CHVs? Do you think it is the same as for other providers, or not as good, or better? Why?
         - Are there advantages of CHVs providing FP services? Do you see any disadvantages?

----------

GENERAL (USERS AND NON-USERS)

1. **Norms and values**
2. In the community, are there certain norms or cultural beliefs in your community about using FP methods?
3. What are some examples of such norms and values?
   - Probe:
     - What about youth who are not married or in union, are they supposed to use FP methods?
     - What about couples married or in union who don’t have children, are they supposed to use FP methods?
     - And couples with already a few children?
     - Other
4. What do you yourself think of such norms and values? Do you sometimes feel different about this or do you mostly agree?
5. What role does religion play when it comes to FP services in your community?
   - Probe for what the religious leaders tell say about FP
6. In your community and culture, are there norms and customs that are likely to support or hinder your own use of FP services? Give examples?
7. In families or the community, are there people that help or hinder, when you want to use FP methods?
   - Probe for examples; support or hindrance from parents, partners, community leaders, clinic staff, etc.
   - Probe: are friends likely to influence your decision to take up FP services? How, who, when?
8. Have you ever come across FP topics being discussed in the media? Are such discussions likely to influence your decision-making to use FP services?
9. How do you think the community can be more involved in providing family planning services to the youth?

SSI-2: Topic guide for CHVs interviews (M/F, married or in union/not married or in, urban/rural

| CODE |  |
| --- | --- |
| Date |  |
| Moderator |  |
| Recorder |  |
| Transcriber |  |
| Venue/location (county, CU) |  |
| Start time |  |
| End time |  |
| Duration |  |
| General comments by the moderator and recorder | How was the process? Was it difficult/easy to manage? Why? Were participants (un) comfortable? Why? |

After reading the consent form and getting their consent, fill in the following table:

| Job title |  |
| --- | --- |
| Years of service |  |
| Age – in whole years |  |
| Sex -M/F |  |
| Marital status  –married/in union  -unmarried/ not in union |  |
| No. living children |  |
| Education  -none  -up to completed primary  -up to completed secondary  -higher educ |  |
| Ethnicity |  |
| Religion |  |
| Active as CHV since how many years  -in whole years |  |

Questions

**Role as CHV**

1. How were you recruited as community health volunteer? Who initiated contact; how did you come to learn about it?
2. Where were you recruited: public Barasa, health facility, by CHC
3. What were the selection criteria: education background, age, gender, religion, other

- Probe: training on FP (knowledge and skills)?
- Probe: capacity to engage with the community on FP and SRH issues?

1. How much time do you spend as CHV every day/week? What are your daily tasks?
2. What attracted you to this job?
3. What do you like and what don’t you like about the job?
   - Probe: support from community, support from the CHMT and facility health workers, the workload

**Youth and FP services in general**

1. Do youth [= 18-24 years] sometimes use FP methods? Why?
2. What type of methods do youth prefer and why?
3. Where and from whom do the youth normally get FP methods from?
   - If needed, probe for facility and person: local government clinic (nurse, other?), local pharmacy/drug seller, private clinic, other
4. Why do youth go to that specific facility/provider for these services?
   - Probe for reasons
5. When youth go for FP services, what type of service are they looking for?
   - Probe: general information on method(s), counselling on what is best for their situation, obtaining the method of their choice
6. What are the issues that make the youth consider going for the FP services or not?
   - Probe for:
     1. Enabling environment that is or is not friendly to the interest of the youth
     2. Youth risk of being stigmatized/isolated by peers
     3. Societal/community norms and values (community see youth as not qualified to access community services)
     4. Individual norms and beliefs (community traditional beliefs)

**CHV role in FP services in general and for youth**

1. What is your role as CHV in the provision of FP services in the community? What kind of FP services do you offer?
   - Probe for: general information on method(s), counselling on what is best for their situation, FP methods
2. To what groups do you offer these services?
   - First wait for answers, ask: ‘any other groups’?
   - Then if needed probe for: married couples? Single women/men? Youth? Other?
3. What type of FP methods do you offer to youth? Are these the methods that youth want?
4. What makes you as CHV more relevant to provide FP services to the youth, both male and female?
   - Wait for answers, ask ‘what else’
   - Then if needed, probe for other CHV characteristics: sex, age, marital status, education level, training receive don FP (knowledge and skills)
   - Probe for how CHV services are different/better than services in facilities elsewhere?
5. What do you consider as helpful or hindering factors for a CHV to provide FP services to youth?
   - Wait for spontaneous answers
   - Then probe for provider-side issues:
     - Range/types of FP methods offered in general
     - Any stock outs of preferred FP methods
     - Availability of educational materials for FP for reference
     - Distance, transport
     - Cost
     - Other?
   - And probe for client/community-side issues:
     - Support from community leaders
     - Support from parents
     - Interest from youth themselves
     - Other?
6. What issues affect your support to FP services for youth? What support do you require to deliver more effective FP services to the youth?
   - Wait for spontaneous answers, then if needed probe for:

Health service side

- - Training on FP services
  - Availability/access to the FP commodities
  - Supervision from Health Care workers (CHEWs, nurses)
  - Task clarity on provision of FP services by CHVs
  - Mentorship and coaching
  - Feedback on services provided by CHVs

Clients/community side

- - Community support/norms
  - Other?

**Norms and values**

1. Do you feel it is good to offer FP services to youth? Why yes/no?
2. Would you say that there are certain norms or cultural beliefs in your community about using FP methods? What are some examples of such norms and values?
   - Probe:
     - What about youth who are not married or in union, are they supposed to use FP methods?
     - What about couples married or in union who don’t have children, are they supposed to use FP methods?
     - And couples with already a few children?
3. What do you yourself think of such norms and values? Do you sometimes feel different about this or do you mostly agree?
4. In your community and culture, are there norms and customs that are likely to support or hinder use of FP services? Give examples?
5. And for youth: are there norms and customs that are likely to support or hinder use of FP services by youth?
6. Do you feel prepared and comfortable to engage with the community on FP and SRH issues?

SSI-3: Topic guide for key informants type 1 - interviews with Community Leaders

| CODE |  |
| --- | --- |
| Date |  |
| Moderator |  |
| Recorder |  |
| Transcriber |  |
| Venue/location (county, CU) |  |
| Start time |  |
| End time |  |
| Duration |  |
| General comments by the moderator and recorder | How was the process? Was it difficult/easy to manage? Why? Were participants (un) comfortable? Why? |

After reading the consent form and getting their consent, fill in the following table:

| Job title |  |
| --- | --- |
| Years of service |  |
| Age – in whole years |  |
| Sex -M/F |  |
| Marital status  -married/in union  -unmarried/ not in union |  |
| No. living children |  |
| Education  -none  -up to completed primary  -up to completed secondary  -higher educ |  |
| Ethnicity |  |
| Religion |  |

Questions

1. What do you do as a leader in this community?
2. At the community level, who has the decision about the number of children in the family? (Probe: Husbands? Mother-in-laws? Religious or community leaders? Co-wives? Others?)
   - Who makes decisions about the spacing of births?
   - How are these decisions made?
3. According to you, what are the reasons for:

- Having many children?
- Having few children?
- Waiting a certain amount of time between pregnancies?

1. What is your opinion about discussing the FP service for the at a community gatherings?
2. Do different categories of leaders discuss FP services for the youth in your community? Probe for
   - Provincial administration
   - Politicians
   - Religious leaders
   - Clan elders
3. What do you know about the FP services uptake at community? Probe for
   - - Service providers
     - Types of FP commodities
     - Policies and Regulations on FP services
     - Availability/Access of FP services
4. Are you aware whether the youth (Married/unmarried) are provided with FP services?
   - If yes, who provides them with these services?
   - Probe for CHV, Nurse, Social Workers, Peers, Clinician/Doctors
5. What kind of support do you think CHVs require to provide FP services in the community?
   - Training on types of FP services
   - Supervision from Health Care workers(CHEWs, Nurses)
   - Protection by law or policy
   - Mentorship and coaching

SSI-4: Topic guide for key informants type 2 - interviews with Youth Leaders of selected Youth Groups (M/F)

| CODE |  |
| --- | --- |
| Date |  |
| Moderator |  |
| Recorder |  |
| Transcriber |  |
| Venue/location (county, CU) |  |
| Start time |  |
| End time |  |
| Duration |  |
| General comments by the moderator and recorder | How was the process? Was it difficult/easy to manage? Why? Were participants (un) comfortable? Why? |

After reading the consent form and getting their consent, fill in the following table:

| Job title |  |
| --- | --- |
| Years of service |  |
| Age – in whole years |  |
| Sex -M/F |  |
| Marital status  -married/in union  -unmarried/ not in union |  |
| No. living children |  |
| Education  -none  -up to completed primary  -up to completed secondary  -higher educ |  |
| Ethnicity |  |
| Religion |  |

Questions

**Perceptions and preferences of youth on access to FP services from CHVs and other providers**

1. What do you understand by the term Family Planning services?
2. How would you describe the knowledge/ awareness among the youth in this community on family planning?

- How would you compare the knowledge/awareness among men and women youth?

1. Where do the youth in this community access family planning services and methods?
2. Who do you think the youth prefer to provide them with FP services from?
   - Probe for reasons why - CHVs, Health Care Workers, Pharmacist
3. In your opinion, what are some of the **factors (both positive and negative)** that influence the uptake of family planning services by youth in this community
   - Probe for societal norms/beliefs on FP - acceptability (culture, religion, peers)
4. In your opinion, what are some of the **personal factors** that may help or hinder youth from accessing FP services as an individual*?*
   - Probe for examples; hindrance from parents, partners, autonomy, belief, peers etc.
5. Have you ever heard of family planning being discussed in the media?
   - Probe on the media type they access more often
6. Are such discussions likely to influence youth decision to take up FP services?
7. What is your opinion on the cost of family planning services? Are they affordable?

**Enabling environment for CHVs in provision of FP services to the youth**

1. Are you aware of any polices, guidelines on family planning services in Kenya? If yes, please specify?

- Do these polices cater for the needs of the youth? In what ways?

1. In your opinion, do you think the service providers (CHVs, Health Care Workers or Pharmacists) are well trained and equipped to provide FP services to the youth?

- Probe for what needs to be improved? (supervision, task clarity, workload, service delivery, availability of job aids for CHVs)

1. What are your perceptions about the working conditions, or place of service delivery of FP commodities
   - Does it provide confidentiality, ensure privacy?
   - Is the place accessible?
2. What are some of the social cultural and religious factors that may hinder uptake of family planning services among the youth in this county?
3. How are the family planning services and commodities managed within this county?

- Probe for distribution of FP commodities, availability of transport

1. Are the family planning services available (currently being provided in this county) able to meet the community needs (variety of FP choices available, etc.?)
2. In your opinion, what are some of the recommendations for improving uptake and accessibility of family planning services to the youth in this community?
   - Probe for training youth CHVs, peer to peer influence

SII-5: Topic guide for key informants type 3 - interview with Coordinators & Managers

(RH coordinator, CHS focal person, Chief nursing officer, CHEWs/CHAs, County pharmacists, NGOs, Ward administrators, Health care workers - from linked facility, HRIO – Health Records Information Officers)

| Date |  |
| --- | --- |
| Name of facility |  |
| Moderator |  |
| Recorder |  |
| Transcriber |  |
| Start time |  |
| End time |  |
| General comments by the moderator and recorder | How was the process? Was it difficult/easy to manage? Why? Were participants (un) comfortable? Why? |

After reading the consent form and getting their consent, fill in the following table:

| Job title |  |
| --- | --- |
| Years of service |  |
| Age – in whole years |  |
| Sex -M/F |  |

Questions

- 1. What is your role in this institutions in relation to the agenda of family planning access and uptake among the youth at the community level?
  2. What policy guidelines either national or county support the work of CHVS in relations to FP?
  3. Are these guidelines available and in use by health workers?
- Probe for samples of the same and if they have been disseminated
  1. What is the level of implementation of the above stated guidelines? (Task shifting policy policies, Technical module for CHVS& Family planning)
- Probe for the specific tasks on Family planning of CHVS as stipulated in the Task Policy
  1. What is the level of engagement of other health workers in the implementation of the task shifting policy? How do they support the role of CHVs for FP services?
  2. What choice of methods are CHVs supposed to offer to youth? Are these methods always available? (stockouts) Do youth ask for methods that the CHVs are not supposed to offer or that are not usually available?
  3. What supporting communication materials (job aids) are available for the CHVs in terms of FP communication to the youth to enable perform their task better?
  4. What are supporting resources available to enable CHVs reach out to the youth within the community? (Transport)
  5. Are CHVs well-motivated for their job and for providing FP services? Do they receive any kind of incentives (general and for FP services)?
  6. How do the CHV access the youth within the community to offer FP services?
  7. What are the mechanisms for distribution of the FP commodities at the community level?
  8. How are the CHV kits constituted and replenished?
  9. What are the specific FP commodities available and how are stock outs monitored
  10. What is the capacity requirement for the CHVs to be able to offer FP services to the youth at level one?
- Type of training in relations to FP and frequency
- Referral of clients/youth to other staff if needed
  1. What technical support/mentorship do other health care workers offer to the CHVs? (CHEWS/CHAs)
  2. What are the available mechanisms to measure the performance of CHVs in terms of FP uptake and access at level one? ((CHRIO) Probe for MOH Reporting tools and what data they capture)
  3. How is FP data uploaded to the system for informed decision making?
  4. What are the available supportive supervision mechanisms to check on quality of the services offered by CHVs? (CHEWs/CHAs)
  5. How is this data on service delivery of FP generated and transmitted to the system?
  6. What are the feedback mechanisms in place? (Probe for Community Based Health Management Information system and presence of community dialogue and action days)
  7. What performance appraisal mechanisms are available to measure the performance of CHVs in relation to FP services? (Probe further which ones are available for use)
  8. What major strengths and challenges are encountered in service delivery of FP commodities to the youth by CHVs?
  9. In your opinion, how can these challenges be overcome?
  10. What is the role of CHVs in provision of FP services to the youth? In theory? And in practice?
  11. What type of FP services are administered by CHVs to the youth?
  12. What is the perception of health workers about CHVs offering FP services to the youth? (CHEW)
  13. What is the community’s perceptions in regard to FP services offered by CHVs to the youth? (CHEW)
